# Evaluation of the anti-nociceptive effects of morphine, tramadol, meloxicam and their combinations using the tail-flick test in rats

**Published:** 2015-12-15

**Authors:** Mehrzad Foroud, Nasser Vesal

**Affiliations:** Department of Veterinary Clinical Sciences, School of Veterinary Medicine, Shiraz University, Shiraz, Iran.

**Keywords:** Anti-nociception, Meloxicam, Morphine, Rat, Tramadol

## Abstract

The purpose of the present study was to evaluate anti-nociceptive effects of morphine, tramadol, meloxicam and their combinations in rats. Seventy male Wistar rats were divided into seven equal groups and randomly assigned to receive intraperitoneal saline (S) (control group, 1.0 mL kg^-1^), morphine (MO) (4.0 mg kg^-1^), tramadol (TR) (12.5 mg kg^-1^), meloxicam (ML) (1.0 mg kg^-1^), tramadol- morphine (TR-MO), meloxicam-morphine (ML-MO) and meloxicam-tramadol (ML-TR) at the same doses. Anti-nociception was evaluated using tail flick latency (TFL) test at 45, 60, 75, 90 and 120 min after drug injection. The TFL was significantly higher in TR and MO groups compared to S group for 90 and 120 min, respectively. No significant change in TFL from baseline values was observed at all time points in ML group. Among rats that received combination of analgesics, those that received TR-MO had significantly greater TFL. There was no significant difference in TFL between ML-TR and ML-MO groups. In conclusion, TR, MO and their combination all provided acceptable anti-nociceptive effects in rats. Meloxicam at the given dosage (1.0 mg kg^-1^) did not demonstrate any anti-nociceptive effect when evaluated by TFL test.

## Introduction

A variety of drugs are available for analgesic therapy in animals. Opioids, tramadol and non-steroidal anti-inflammatory drugs (NSAIDs) are used routinely in veterinary medicine. Opioids have a high analgesic efficacy as well as some undesirable properties including sedation, respiratory depression, decreased gastrointestinal motility and development of analgesic tolerance after repeated administration.^1^^,^^2^ Morphine has primary activity at mu receptors with some activity at kappa and delta receptors. It is used for relief of moderate to severe pain of different etiologies.^2^ Tramadol, a centrally acting analgesic, provides pain relief by means of its mu agonist activity and reuptake inhibition of norepinephrine and serotonin. This dual mode of action distinguishes it from opioids. It has fewer side effects than opioids and is not a controlled drug in some countries.^2^^,^^3^ Meloxicam, a NSAID, is used as an analgesic in human and veterinary medicine. Like other NSAIDs, meloxicam exhibits analgesic and anti-inflammatory activities probably through inhibition of cyclo-oxygenase (COX), and the inhibition of prostaglandin synthesis. It is considered COX-2 preferential.^2^^,^^4^ The NSAIDs have been shown to provide sufficient analgesia for acute postoperative pain in rats. However, some adverse effects such as gastrointestinal ulceration and vomiting are probable. The NSAIDs are not categorized as controlled drugs.^2^^,^^3^

Many of the adverse effects of the analgesic drugs are dose-dependent. Therefore, the combinations of different classes of drugs are commonly used to control pain.^1^^,^^5^^,^^6^ Multimodal or balanced analgesia involves the use of a combination of analgesic drugs from different classes to yield additive analgesic effects. Combining the analgesic drugs can decrease the side effects of each drug and increase the efficacy.^3^^,^^7^

The tail flick test is a thermal latency assay that has been extensively used to evaluate the anti-nociceptive effect of various drugs given systemically in both rats and mice.^8^^,^^9^ The aim of present study was to compare the anti-nociceptive effects of intraperitoneal (IP) single dose of morphine, tramadol and meloxicam when used alone or in combination, using tail flick test in the male Wistar rats. 

## Materials and Methods

**Animals. **Seventy male 250-300 g Wistar rats were used in a blinded, randomized study. Rats were housed in a temperature of 21 to 22 ˚C and lighting interval of 12-hr light/12-hr darkness in the controlled environment. Standard laboratory pellet food and tap water were available *ad libitum* throughout the study. This experimental study was approved by the Institutional Animal Care and Use Committee.

**Procedures. **Rats were randomly assigned into one of seven groups (10 rats per group) for IP administration of the following drugs: 1.0 mL kg^-1 ^saline (S, Shahid Ghazi Pharmaceutical Co., Tabriz, Iran) (control group), 4.0 mg kg^-1 ^morphine (MO; Darou Pakhsh, Tehran, Iran),^8^^,^^10^^,^^11^ 12.5 mg kg^-1 ^tramadol (TR; Tehran Chemie Pharmaceutical Co., Tehran, Iran) ^12^, 1.0 mg kg^-1 ^meloxicam (ML; Boehringer Ingelheim Vetmedica Gmbh, Ingelheim, Germany) ^13^, tramadol-morphine (TR-MO), meloxicam-morphine (ML-MO) and meloxicam-tramadol (ML-TR) at the same doses. All drugs were diluted with sterile saline and a final volume of 1 mL kg^-1^ was administered IP into the right caudal abdominal quadrant in each rat. Identical coded syringes were prepared by a person not involved in the study. Each rat received only one treatment.

**Fig. 1:**
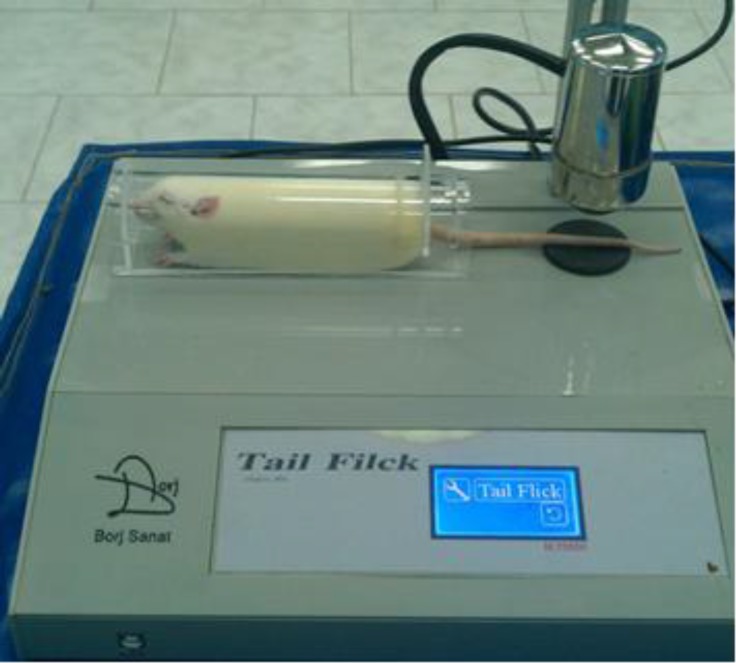
Use of a plexiglass cylinder to restrain the rat during the tail-flick test

Anti-nociception was assessed by the tail flick latency (TFL) test using an automatic analgesiometer (Tail flick; Borj Sanat, Tehran, Iran), (Fig. 1). The TFLs were measured as the time between tail exposure to radiant heat and tail withdrawal. An intensity setting of 25 on a scale of 1 to 100 and a cut-off time of 15 sec, to prevent tissue damage, was used throughout the study.^8^ The light beam was focused on the rat’s tail about 4 cm from the tip. The radiant intensity was adjusted to give a baseline TFL of 4 to 6 sec and animals with a baseline TFL below 4 or above 6 sec were excluded. Baseline latency time was derived from the mean of two TFL tests with an interval of 10 min between readings. Rats were injected with the drugs, as described above, 15 min after the baseline measurement. The TFL tests were performed at 45, 60, 75, 90 and 120 min after injection. The mean of two consecutive readings with an interval of 1 minute was recorded as the TFL value at the mentioned time points. The same investigator assessed the TFL in all groups and was unaware of the treatment given.

**Data and statistical analysis. **Anti-nociception was expressed by mean percentage maximum possible effect (%MPE). The %MPE was calculated using the following formula:

% MPE =Post-drug TFL – Baseline TFL5 sec – Baseline TFL×100

The baseline TFL was compared between the groups using one-way analysis of variance (ANOVA). Differences in MPE among the groups at each time point were analyzed by a mixed model of variance with drug, time and drug-by-time interaction as fixed effects and animal within treatment as a random effect followed by Bonferroni multiple comparisons. The analysis of the overall (45-120 min) MPE between the groups were performed using ANOVA followed by Tukey’s test. The data were analyzed using SPSS (version 17; SPSS Inc., Chicago, USA). A *p*-value of less than 0.05 was considered statistically significant. All data are presented as mean ± SD.

## Results

**Fig. 2:**
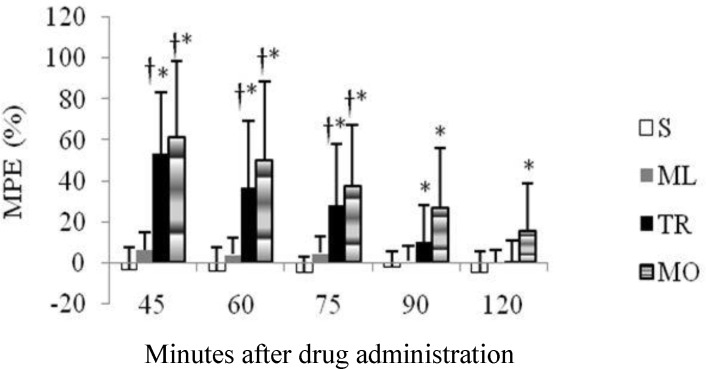
Anti-nociceptive effects of intraperitoneal administration of saline (S), meloxicam (ML), tramadol (TR) and morphine (MO) on tail-flick latency (TFL) in rats (10 rats per group). The TFL (mean ± SD) is expressed as %MPE.

The baseline TFL was 4.64 ± 0.62 and there was no significant difference between the groups. For the first 90 min after injection, the mean of MPE was significantly higher than control group in TR group, whereas MO significantly increased MPE at all time points following injection. Meloxicam did not produce a significant change in the TFL from baseline over the 120 min trial (Fig. 2). Rats received either TR or MO had significantly higher MPE compared with ML group from 45 to 75 min. The TFLs produced by TR and MO did not have any significant difference at any time points (Fig. 2). 

**Fig. 3:**
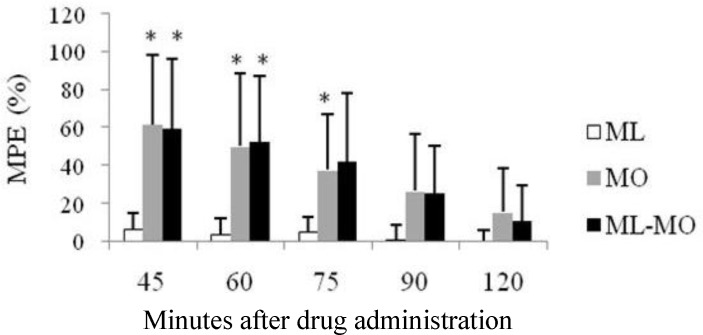
Anti-nociceptive effects of intraperitoneal administration of meloxicam (ML), morphine (MO) or their combination (ML-MO) on tail-flick latency (TFL) in rats (10 rats per group). TFL (mean ± SD) is expressed as %MPE

**Fig. 4:**
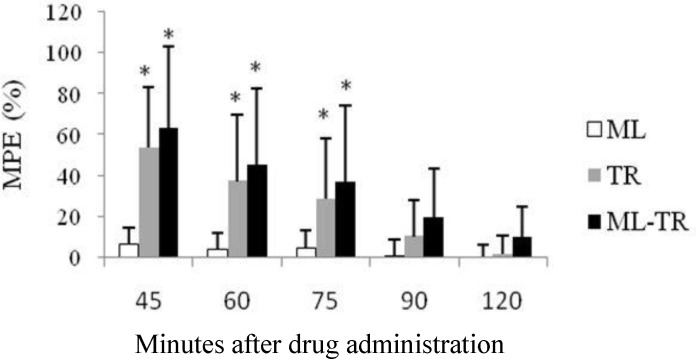
Anti-nociceptive effects of intraperitoneal administration of meloxicam (ML), tramadol (TR) or their combination (ML-TR) on tail-flick latency (TFL) in rats (10 rats per group). TFL (mean ± SD) is expressed as %MPE.

**Fig. 5.**
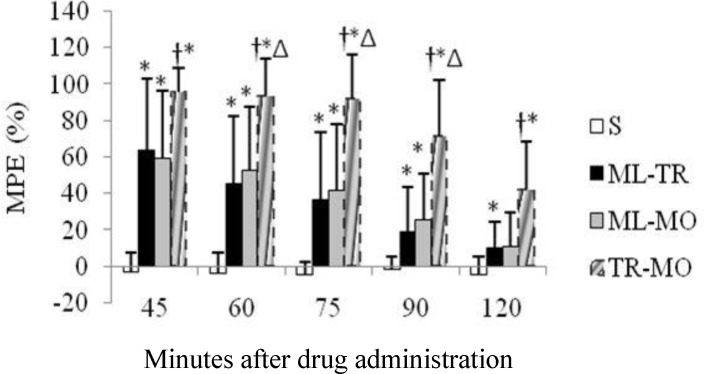
Anti-nociceptive effects of intraperitoneal administration of saline (S), meloxicam-tramadol (ML-TR), meloxicam-morphine (ML-MO) and tramadol-morphine (TR-MO) on tail-flick latency (TFL) in rats (10 rats per group). TFL (mean ± SD) is expressed as %MPE.

The co-administration of MO or TR with ML produced latencies that did not differ from those of MO and TR alone (Figs. 3 and 4). Among groups that received combination of analgesic drugs, those that received TR-MO had significantly higher MPE compared with ML-TR group (at the 60-, 75- and 90-minutes time points) and ML-MO group (at all time points), (Fig. 5). There was no significant difference in the mean TFL between ML-TR and ML-MO groups (Fig. 5).

* indicates significant difference compared to saline control group (*p *< 0.05), and ^†^ indicates significant difference compared to ML group (*p *< 0.05).

* indicates significant difference compared to ML group (*p *< 0.05).

* indicates significant difference compared to ML group (*p *< 0.05).

**Fig. 6:**
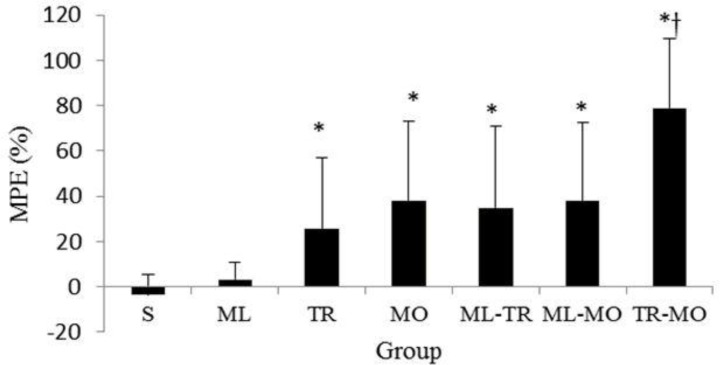
Overall (45-120) %MPE in saline (S), meloxicam (ML), tramadol (TR), morphine (MO), meloxicam-tramadol (ML-TR), meloxicam-morphine (ML-MO), and tramadol-morphine (TR-MO) treatments. The TFL (mean ± SD) is expressed as %MPE.

The peak anti-nociceptive effect of the drugs was observed at 45-min time point and the mean of MPE decreased over time within all treatment groups (Figs. 2 and 5). Analyzing the overall (45 to 120 min) MPE between the treatments showed that anti-nociceptive efficacy of TR-MO combination was significantly superior to other groups, whereas the ML group was devoid of any anti-nociceptive activities (Fig. 6).

Two rats in TR-MO group exhibited signs of sedation (reduced cage activity and slow movement) and were reluctant to move their tails away from the light beam at the 45-90 min time points. No adverse effects related to the treatments (anorexia, pica behavior or death) or exposure to the thermal stimulus (tail skin damage) were observed during 7 days after the completion of the experiment.

## Discussion

The results of the present study demonstrated that IP administration of TR-MO combination provides higher anti-nociceptive effects compared with MO, TR or ML alone, and ML-TR or ML-MO combinations. Meloxicam, at the given dose (1.0 mg kg^-1^), had no significant effects on the TFL test in rats.

Tail flick test is an objective and quantifiable measure of pain that has been used for assessing anti-nociceptive activity of drugs. This test can involve both spinal and supra spinal structures, depending on the intensity of the radiant heat stimulation.^14^ In the present study, the setting of the analgesiometer was adjusted to elicit a reaction within 4 to 6 sec which converts the tail flick reflex from a purely spinal one to a more complicated reaction which involves higher neural structures. This setting could give the tail flick test the privilege for measuring anti-nociceptive efficacy of different classes of drugs.^8^^,^^14^

Morphine retained its anti-nociceptive efficacy through-out the study, as compared to saline group. Single dose of morphine (3 to 10 mg kg^-1^) has been reported to provide analgesia for up to two hours in rats which was similar to our results.^10^^,^^15^^,^^16^ Morphine is considered the drug of choice for alleviating moderate to severe pain and the dosing interval of 2 to 3 hr has been suggested in rats.^16^

Analgesic and anti-hyperalgesic effects of tramadol have been reported previously in rats.^12^^,^^17^ Cannon *et al.* evaluated the efficacy of tramadol at four dosages (4.0, 12.5, 25.0 or 50 mg kg^-1^) and concluded that tramadol, at the dose of 12.5 mg kg^-1^, provides effective analgesia for approximately 60 min following IP administration.^12^ The anti-nociceptive effect of tramadol was observed for about 90 min in the present study. 

Meloxicam is frequently used as an analgesic in laboratory animals.^13^^,^^18^^-^^20^ In the present study, administration of meloxicam (1.0 mg kg^-1^, IP) did not increase the TFL at any time points. It has been reported that meloxicam (1 to 3 mg kg^-1^, intravenous) does not reduce isoflurane minimum alveolar concentration (MAC) in the rat.^4^ Higher doses of Meloxicam (13.0 mg kg^-1^, IP) may possess anti-nociceptive activity in tail flick test in mice;^21^ however, meloxicam at the dose of 5.8 mg kg^-1^ did not change nociceptive thresholds to a thermal stimulus applied on the hindpaw of normal rats.^22^ The NSAIDs were inefficient to increase the pain threshold in threshold escape paradigms such as tail flick and hot plate tests.^23^ It has been reported that meloxicam attenuated laparotomy induced pain in rats and mice.^13^^,^^20^ The anti-inflammatory activity of meloxicam is mediated by inhibition of the COX enzymes and blocking the synthesis of prostaglandins at the site of tissue injury.^2^ Therefore, the administration of NSAIDs would be more effective when inflammation is expected. 

Multimodal analgesia refers to the application of different classes of analgesic drugs to target different points in pain pathways and gain additive or synergistic effects. This approach reduces the potential for undesirable side effects associated with higher doses of individual drugs.^3^^,^^24^ In the current study, combination of MO and TR provided superior pain relief, compared with either drug alone. Improved analgesia has been reported following addition of TR to MO after abdominal surgery in humans.^25^ Additive but not synergistic interaction of MO and TR has been observed in mice using hot plate test.^5^ Considering the use of fixed doses of drugs in our study, distinguishing synergistic from additive effects was impossible. Sedation was seen in two rats following administration of TR-MO combination. Sedation has been reported after administration of tramadol at doses greater than 15 mg kg^-1^ in rats.^12^^,^^26^ However, no side effects have been observed following addition of TR to MO in humans.^25^

The combination of opioids and NSAIDs has been successfully used in human and veterinary patients and it has been accepted that NSAIDs improve the analgesic effect of opioids.^3^^,^^8^ In the present study, ML-MO combination showed significantly greater TFL time compared with control and ML groups; however, addition of ML did not result in potentiating of MO analgesic effects. Intravenous meloxicam (1 to 3 mg kg^-1^) did not potentiate the morphine-induced decrease of isoflurane MAC in the rat.^4^ A previous study in rats found that dipyrone potentiates anti-nociceptive effects of MO in tail flick.^8^ Synergistic interaction between hydrocodone and ibuprofen (a non-selective cyclooxygenase inhibitor) has been reported previously in mice, using tail flick test.^27^ In contrast, no additive or synergistic interaction between morphine and ketorolac (a non-selective cyclooxygenase inhibitor) was found in the rat immersion tail flick.^28^ This disparity between the studies may be due to species differences (mice *vs.* rat), different types of pain (radiant heat *vs.* hot water immersion) and different settings for pain assessment (i.e., intensity of thermal stimulus applied).^8^ A recent study demonstrated that the interactions of these drugs are markedly dependent upon which NSAID/opioid are examined.^29^ Ibuprofen and naproxen potentiated the analgesic action of hydrocodone and oxycodone but neither aspirin nor ketorolac influenced hydrocodone action in mouse tail flick test. In the same study, ibuprofen did not potentiate fentanyl or morphine analgesia.^29^

Co-administration of TR and ketorolac has produced analgesic effects greater than that observed after individual treatment in a rat model of arthritic pain^1^, whereas behavior based assessment of the laparotomy pain in rats showed similar analgesic effects of tramadol, carprofen and their combination.^3^ In the present study, ML did not show any significant potentiating effect when added to TR. The NSAIDs possess different capacities to induce the inhibition of COX. Meloxicam, as a preferential inhibitor of inducible COX-2, may be less effective in the tail flick test as a somatic pain model without inflammation.^9^

In conclusion, the results of present study indicate that TR and MO provide acceptable anti-nociceptive effects in rats. Combination of TR and MO showed strong anti-nociceptive effects. However, this combination may result in some sedation at the dose given here. Although ML at the given dosage (1.0 mg kg^-1^) did not demonstrate any anti-nociceptive effect when evaluated by TFL test, it may still be useful in providing postoperative analgesia.^13^^,^^19^ Dose-response studies are required to determine the type of interaction (additivity or synergy) between MO, TR and ML in rats.
